# Editorial: Be positive about the negative in pharmacology: clinical studies in renal pharmacology 2022

**DOI:** 10.3389/fphar.2023.1236750

**Published:** 2023-06-16

**Authors:** Narayan Prasad, Brijesh Yadav

**Affiliations:** Department of Nephrology, Sanjay Gandhi Postgraduate Institute of Medical Sciences, Lucknow, India

**Keywords:** IgA nephropathy, diabetic kidney disease, VEGF–vascular endothelial growth factor, podocyte, CYC (cyclophosphamide)

For the progress of science, it is imperative to publish robust, reliable, and replicable data (Nosek et al., 2022). In this scenario, publishing negative and positive results becomes equally important. However, increasing pressure from peers, funding agencies, citation counts, recruiters, publishers, and the pressure to get published or perish forces researchers to compromise the reporting of the factual findings in many instances. The researchers tend to publish only the positive results that attract higher citations, audiences, generate newer hypotheses and funding over the negative results (Fanelli, 2012). This results in publication bias and diverts the research in particular areas, leaving the alternate sides neglected, although that also deserves to be investigated in actuality and may be equally important too. It hinders the development of alternative hypotheses and strategies to deal with specific problems (Ropovik et al., 2021).

It has also been observed that publishing negative results is quite difficult, attracts fewer citations, and is treated more as a technical failure of experiments rather than negative results (Catalini et al., 2015). In a study by Daniele, it was found that 85% of studies across the different disciplines published only positive results between 1997 and 2007 (2), suggesting a gradual exodus of negative results from scientific publications. Sometimes results remain inconclusive because of limitations of techniques, which encourage future researchers to use better techniques to get the result, and that should be treated as having a positive impact otherwise. Researchers often publish only positive results that prove their hypothesis, despite having some negative results. Sometimes results are not consistently achieved within the stipulated sample size, and researchers increase the number of biological replicates to achieve statistical significance without revealing the actual number of biological replicates used in experiments. Due to the pressure of publication and corroboration with previous findings, a few researchers manipulate data in line with reputed publications. Many a time, it becomes hard for them to publish if results contradict or find contradictory findings published in high-impact factor journals and are cited by many authors, despite the well-accepted fact that results published in high-impact factor journals are also not reproducible in other researcher’s labs (Yong, 2012), (Begley and Ioannidis, 2015).

In the scientific community, research moves forward with the communication amongst the labs, and publication is an important bridge for this communication. The publication series *Be Positive with negative results in Pharmacology* emphasizes the importance of publishing both positive and negative results in medical research. It also highlights the fact that the publishing negative results is equally important as publishing positive results. Moreover, for the negative results of a given study, there is need for validation by other studies. This saves the time, finances, resources, and manpower of the other future researcher, for whom the results are never going to be achieved, and there is no need for repetition of those experiments by the prospective researcher (Baker, 2016).

Ensuing the importance of negative results in the progress of medical research, in this special edition *Be positive with negative results in pharmacology*, four original research articles that challenged the previous assumptions and investigated the contradictory effects of pharmacological interventions in managing renal diseases has been published. Each study addresses a specific aspect of treatment and provides valuable insights into the efficacy and potential risks, benefits associated with the interventions.

In the first study, Cheng et al. and colleagues examined the effects of oral sodium bicarbonate supplementation in patients with advanced renal failure. The use of sodium-based interventions is a concern for patients with advanced renal failure, as sodium leads to water retention and edema and may be associated with uncontrolled hypertension and cardiac failure. Despite concerns about sodium intake in these patients, the researchers found that sodium bicarbonate therapy had beneficial effects, including equal dialysis start timing, lower risk of major adverse cardiovascular events, pulmonary edema, and mortality. This suggests that sodium bicarbonate supplementation can be beneficial for this patient population. It is possible that sodium with chloride may have differential effects on hypertension and water retention, compared to sodium with bicarbonate preparation (Luft et al., 1988; Luft et al., 1990; Brar et al., 2008).

The treatment of IgA Nephropathy with immunosuppressive therapy is still controversial and evolving. In the second study, Luo et al. and colleagues, evaluated the impacts of renin-angiotensin-aldosterone system blockers with different combination of immunosuppressive medicines on the progression of IgAN with isolated hematuria. The researchers found that isolated hematuria reflects IgAN progression, and podocyte cells are the main targets. The study also highlighted the role of oxidative stress in podocyte cells and the positive effects of immunosuppressive therapy on reducing oxidative stress and cellular damage. The cellular crescent formation and hematuria also decreased significantly, and chronicity index increased, and IgA, C3 deposition remained unchanged. The authors observed a marked improvement in podocyte mitochondria count, reducing podocyte fissure gap and reducing oxidative stress biomarkers in the second biopsy after immunosuppressive therapy. More importantly after treatment with immunosuppressive, no side-effects were observed, that highlights the safety of treatment combinations.

In another study on IgAN in this special series, Luo et al. and colleagues evaluated the use of sequential immunosuppressive therapy in treating IgAN. Authors compared the outcomes of patients on standard supportive therapy, prednisolone only group (P-group), prednisolone combined with cyclophosphamide followed by mycophenolate mofetil (P + CTX), and prednisolone combined with mycophenolate mofetil (P + MMF). They observed that the estimated glomerular filtration rate (eGFR) was higher at 6 and 24 months in the P, P + CTX, and P + MMF groups compared to the supportive group. At 24 months, the eGFR was higher in the P + CTX group than in the P + MMF group. At 24 months, the effective remission rate was higher in the P + CTX group. The effective remission rate was also higher at 12 months in the P group compared to the standard supportive group. However, at 24 months, the effective remission rate was not significantly different among these groups. This study suggests that a P + CTX treatment may be a therapeutic option for IgAN patients during the early period.

In a study, Xiao et al. and colleagues explored the use of intravitreal vascular endothelial growth factor (VEGF) inhibitor therapy for treating diabetic retinopathy in patients with diabetic kidney disease. Previous observations indicated potential systemic side effects of VEGF inhibitors, including renal side effects such as a rise in serum creatine level, proteinuria, podocytopathy, and thrombotic microangiopathy (Hanna et al., 2020; Izzedine et al., 2014). VEGF is also required for the repair of endothelial cell injury and the neovascularization of the glomerulus and peritubular capillaries. The authors also evaluated the long-term renal outcome in patients with biopsy-proven diabetic kidney disease after VEGF inhibitor therapy. However, one-year renal and patient survival was better in the VEGF-inhibitors users group than non-users. However, after 5 years of follow-up, it was similar between the users and non-users. This study provides valuable insights into the effects of VEGF inhibitors on kidney function.

Overall, the publication series highlights the importance of publishing both positive and negative results to advance scientific knowledge. By sharing negative and inconclusive findings, researchers can prevent unnecessary repetition of experiments, save resources, and foster the development of alternative hypotheses and strategies. This approach can accelerate scientific progress and facilitate effective communication among researchers.
